# Adaptive Radiation along a Thermal Gradient: Preliminary Results of Habitat Use and Respiration Rate Divergence among Whitefish Morphs

**DOI:** 10.1371/journal.pone.0112085

**Published:** 2014-11-18

**Authors:** Kimmo Kalevi Kahilainen, William Paul Patterson, Eloni Sonninen, Chris Harrod, Mikko Kiljunen

**Affiliations:** 1 Kilpisjärvi Biological Station, University of Helsinki, Kilpisjärvi, Finland; 2 Department of Environmental Sciences, University of Helsinki, Helsinki, Finland; 3 Department of Geological Sciences, Saskatchewan, Isotope Laboratory, University of Saskatchewan, Saskatoon, Saskatchewan, Canada; 4 Finnish Museum of Natural History, University of Helsinki, Helsinki, Finland; 5 School of Biological and Chemical Sciences, Queen Mary University of London, London, United Kingdom; 6 Instituto de Ciencias Naturales Alexander Von Humboldt, Universidad de Antofagasta, Antofagasta, Chile; 7 Department of Biological and Environmental Sciences, University of Jyväskylä, Jyväskylä, Finland; University of Shiga Prefecture, Japan

## Abstract

Adaptive radiation is considered an important mechanism for the development of new species, but very little is known about the role of thermal adaptation during this process. Such adaptation should be especially important in poikilothermic animals that are often subjected to pronounced seasonal temperature variation that directly affects metabolic function. We conducted a preliminary study of individual lifetime thermal habitat use and respiration rates of four whitefish (*Coregonus lavaretus* (L.)) morphs (two pelagic, one littoral and one profundal) using stable carbon and oxygen isotope values of otolith carbonate. These morphs, two of which utilized pelagic habitats, one littoral and one profundal recently diverged via adaptive radiation to exploit different major niches in a deep and thermally stratified subarctic lake. We found evidence that the morphs used different thermal niches. The profundal morph had the most distinct thermal niche and consistently occupied the coldest thermal habitat of the lake, whereas differences were less pronounced among the shallow water pelagic and littoral morphs. Our results indicated ontogenetic shifts in thermal niches: juveniles of all whitefish morphs inhabited warmer ambient temperatures than adults. According to sampling of the otolith nucleus, hatching temperatures were higher for benthic compared to pelagic morphs. Estimated respiration rate was the lowest for benthivorous profundal morph, contrasting with the higher values estimated for the other morphs that inhabited shallower and warmer water. These preliminary results suggest that physiological adaptation to different thermal habitats shown by the sympatric morphs may play a significant role in maintaining or strengthening niche segregation and divergence in life-history traits, potentially contributing to reproductive isolation and incipient speciation.

## Introduction

A major task in evolutionary biology is to understand the formation of new species. Adaptive radiation is recognised as a key mechanism in speciation [1]. A central tenet of adaptive radiation is phenotypic adaptation to a specific environment [1]. Here, disruptive selection linked to adaptation to different environments is considered an important mechanism, both with regard to the initial divergence, as well as the maintenance of differences [1]. The final outcome of disruptive selection may lead to the formation of new species via ecological speciation [2]. Speciation research conducted over recent decades has demonstrated that many avian, terrestrial and aquatic species are now considered to have arisen via adaptive radiation and ecological speciation [1]–[3].

Most of this speciation research has focused on ecomorphological divergence, whereas the contribution of thermal adaptations in ecological speciation is generally poorly understood [4]. Many species distributions of closely related species exhibit distinct correlations with ambient temperature rather than ecological niche segregation [5]. Many famous adaptive radiations including Darwin’s finches and *Anolis* lizards occur on volcanic island environments displaying pronounced altitudinal and vegetation gradients, resulting in different temperature and precipitation conditions [6], [7]. However, even in these model systems most of the research has focused on ecomorphological divergence, largely neglecting the potential selective importance of temperature and related thermal adaptations. Such adaptations are likely most important for poikilotherms, such as amphibians, reptiles and fish.

Polymorphic fishes inhabiting post-glacial lakes have become important models for understanding how disruptive selection acts on phenotypic divergence and how it is related to accumulation of reproductive isolation between diverging species [8]–[10]. Across different fish lineages, a common pattern displayed is morphological divergence correlated to specific resource utilization in pelagic-littoral resource axis in lakes [e.g. 11]. In more complex cases, morphological divergence can be extended to a third principal lake habitat, the profundal [12]–[14]. In contrast to morphological divergence, which is well studied, far less is known about physiological adaptations to specific niches. Because freshwater fishes can be divided into cold, cool and warm water adapted species according to species-specific thermal optima, tolerance ranges and habitat selection [15], it is also likely that physiological adaptions should arise early in the divergence process. One of the best experimental studies comes from the salmonid fishes, where the closely related and morphologically similar cisco (*Coregonus fontanea*) and vendace (*Coregonus albula*) from Lake Stechlin in Germany differ in their optimum temperature preferences and standard metabolic adaptations [16]. Beyond this, little is generally known about temperature preferences and metabolic adaptions of polymorphic fish.

Adaptive radiation and ecological speciation is common throughout the natural range of European whitefish (*Coregonus lavaretus* (L.)) [17]–[19]. Whitefish are known for their ability to diverge, allowing the utilisation of pelagic and benthic habitats in lakes, with up to five sympatric morphs in the same lake [13], [17], [20]. Ecomorphological divergence of whitefish into morphs using the three major habitats (i.e. pelagic, littoral and profundal zones) in large, deep lakes is particularly pronounced in northern Fennoscandian lakes [12], [13], [21]. Apart from variation in food resources [22] and basal sources of energy [21], these habitats also differ with regard to their thermal conditions. Thermal stratification of the water column in these lakes means that littoral and pelagic habitats undergo large seasonal changes in water temperature between warm summer months (water temperature range 10–20°C) and cold winter months (0–2°C), when lakes are ice-covered [22]. Profundal fish occupy a cool/cold habitat (2–8°C) throughout the year. Reproductive isolation among whitefish morphs is generally based on spatial and temporal differences in spawning place and time [14], [17], [19].

Whitefish morphs dwelling in these different habitats differ in the number of gill rakers, which is commonly correlated to trophic ecology and habitat use in each of sympatric whitefish morphs and is also a heritable trait [9], [17], [23], [24]. Gill raker number is closely correlated with overall head and body morphology: pelagic morphs are typically fusiform with a higher number of gill rakers, whereas benthic morphs have more robust body shape and a lower number of gill rakers [21], [23]. Much of our understanding of polymorphic whitefish ecology relates to studies conducted during summer [21], [23], [25], and equivalent studies of habitat selection during winter months (i.e. when lakes in these regions are ice covered) are lacking. Furthermore, data regarding lifetime patterns of thermal niche selection from the point of hatching to maturation are not currently available from polymorphic fish.

Such studies of long-term thermal habitat selection could include the use of electronic data storage tags. However, these approaches are not suitable for delicate and small bodied fishes such as pelagic and profundal whitefish morphs. Here, it is more suitable to use natural anatomical recorders from individual fish such as balancing bones (e.g. otoliths), that document information such as secular variation in seasonal metabolism [26], thermal preferences [27], and growth of fish [28]. Furthermore, otolith microchemistry provides a chemical record of the annual changes in temperature related to metabolism that can be used to quantify variation in the life-time thermal niche via stable isotope analysis [29]. Oxygen stable isotope values (δ^18^O) have also been used to calculate temperatures encountered during the lifetime of fishes living in the distant past [e.g. 30]. Furthermore, the inorganic carbon isotope value (δ^13^C) of otoliths is correlated with the specific respiration rate of individual fish [e.g. 26,31,32], and analysed in parallel with δ^18^O values. The use of micromilling techniques facilitates sampling of otoliths at a subannual scale throughout the lifetime of the fish by discretely isolating aliquots of carbonate that may represent days to weeks, depending on the growth rate and size of the otolith [29], [33]. While otolith δ^18^O and δ^13^C values have been successfully used to track the lifetime thermal niche and respiration rates for many marine and freshwater species [32], [34]–[36], we are unaware of any comparative studies of polymorphic species.

In this preliminary study, we were interested in putative differences in lifetime thermal niche selection among individuals of different sympatric whitefish morphs inhabiting a subarctic lake and whether such whitefish morphs displayed ontogenetic thermal shifts. If differences in thermal niche selection are apparent, we were interested in determining any correlative differences in respiration rates among whitefish morphs. Finally, we evaluated the likelihood of different hatching times among whitefish morphs using micromilling of otolith nuclei. According to previous data collected during summer sampling campaigns relating to temperature and habitat selection, the profundal whitefish morph was hypothesized to dwell in the coldest and the most annually thermally stable environment relative to the littoral and pelagic morphs [21], [25]. If the profundal morph is adapted to a cold and thermally stable environment, we predicted that its respiration rates should be both lower and less variable than morphs that inhabit shallower, warmer habitats. Following the results of a previous otolith isotope study conducted on an alpine monomorphic whitefish population in Lake Annecy, France [32], we hypothesized that all whitefish morphs likely undertake an ontogenetic niche shift from a warmer juvenile thermal niche to a cooler adult thermal niche and that in all morphs respiration rates should decrease with age. Because all sympatric whitefish morphs investigated in this study lake display genetic differences and partial reproductive isolation according to neutral nuclear markers [14], [37], we expected to find measurable differences in estimated hatching temperatures among the morphs, reflecting differences in spawning time and inter-morph reproductive isolation.

## Materials and Methods

### Study area and fish fauna

**Figure 1:**
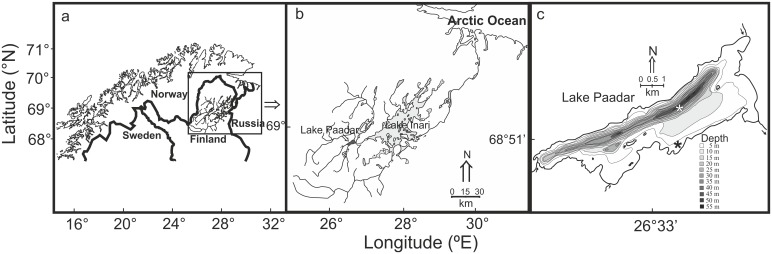
Study area in northern Fennoscandia (a), highlighting the Paatsjoki watercourse (b), and study lake (c). The asterisk in Fig. b indicates the position of water column temperature measurements in 1999–2004 Lake Inari (see Figure 2) and in Fig. c, the position of water column isotope sampling in the pelagic (white asterisk) and littoral (black asterisk) habitats in Lake Paadar (data presented in Figure 3).

Samples were collected from the subarctic oligotrophic (total phosphorus 6 µg/L and total nitrogen 160 µg/L) Lake Paadar (68°52′N, 26°35′E), located in the Paatsjoki watercourse in Finnish Lapland (Fig. 1). The drainage area comprises a mixture of peatland, pine (*Pinus silvestris*) and birch (*Betula* spp.) forest. The surface area of the lake is circa 21 km^2^, the maximum depth is 56 m, with a mean depth of 11.7 m [40]. Proportions of principal habitat types in this lake are littoral 38% and pelagic/profundal 62% [21]. The fish fauna consists of nine species, whitefish, brown trout (*Salmo trutta* L.), grayling (*Thymallus thymallus* (L.)), pike (*Esox lucius* L.), burbot (*Lota lota* (L.)), perch (*Perca fluviatilis* L.), three-spined stickleback (*Gasterosteus aculeatus* L.), nine-spined stickleback (*Pungitius pungitius* (L.)) and minnow (*Phoxinus phoxinus* (L.)) [40]. Whitefish dominate the fish community, contributing ∼90% of the total catch by number [23]. Lake Paadar whitefish stock has evolved into two pelagic and two benthic morphs, each are genetically differentiated from each other [37], according to neutral nuclear DNA (range of F_st_ = 0.02–0.08).

### Sampling

Whitefish were sampled for research purposes only in September 2004 using a gill net series combined of eight gill nets, each 30 m long and 1.8 m high, with different mesh sizes (12, 15, 20, 25, 30, 35, 45 and 60 mm). Gill net series were set in littoral, pelagic and profundal habitats in the evening and collected the following morning. Fish were immediately removed from gill nets, euthanized by cerebral concussion according to the Finnish Animal Conservation Law (32§ 9.8.2013/584) conducted by lead author (KKK) and subsequently placed on ice for measurements in the laboratory. Fishing rights in Finland belong to the land owner according to the Finnish Fishing Law (5§ 27.5.2011/600) and lead author (KKK) get a permit for research fishing from the land owner (Forest and Park Service, permit number 14.5.2004, 1386/5713-2004). No ethical permission is required for described scientific sampling with gill nets according to the Finnish Animal Conservation Law (7§ 28.6.2013/498).

Fish were divided by species (whitefish by morphs) and total length (±1 mm) and wet mass (±0.1 g) were measured from every captured fish. Whitefish were classified into morphs in the field reflecting differences in gill raker, body and head shape [12]. The profundal small sparsely rakered (SSR) benthivorous whitefish morph are small bodied with the lowest relative number (circa 15–20) of short, bent and very widely spaced gill rakers. They also have a characteristic sub-terminal mouth and large eyes positioned high on the head, large fins and a robust body shape [12], [21]. The littoral benthivorous morph, that we refer to as the large sparsely rakered (LSR) morph has an intermediate number (c. 20–30) of short and widely space gill rakers, characteristic whitefish coloration and a terminal mouth. A similar body and head shape is typical for the large densely rakered (LDR) morph, but compared to the LSR morph, gill rakers are more numerous (c. 30–36), longer and more densely spaced. The fourth morph is the densely rakered (DR) whitefish, with a slender fusiform body, superior mouth, and numerous (c. 30–40), fine, long and very densely spaced gill rakers [12], [21]. A size-based subsample (c. 40–50 individuals) reflecting the length distribution of the catch was frozen (−20°C) for more detailed analysis. The main prey items of the different whitefish morphs were collected for carbon and nitrogen stable isotope analyses [21]. Pelagic zooplankton was collected with vertical hauls using 25 cm diameter (50 µm mesh size) plankton net. Littoral (sampling depths 1–3 m) and profundal (10–40 m) invertebrate samples were collected using Ekman grab. Water samples for oxygen and inorganic carbon stable isotopes were collected at the end of July 2012 from pelagic and littoral habitats (Fig. 1). The oxygen isotope values of water in the pelagic zone is unlikely to change considerably despite the time delay between otolith (2004) and water sampling (2012) due to the long residence time of the large study lake and the sampling time in the middle of summer [38], [39]. For inorganic carbon isotopes, there are obviously some seasonal differences between thermally stratified and non-stratified seasons [32] and thus our results from summer thermal stratification should be considered as a snap shot. Temperature and oxygen profiles were recorded at the same time.

### Laboratory work

Whitefish samples were thawed in the laboratory and positioned for photographs taken of the left flank of each fish [21]. The first left gill arch was subsequently dissected and gill rakers were counted under a microscope. The middle gill raker length was also measured for comparison of pelagic and benthic morphs [12]. Scales and sagittal otoliths were removed for the determination of age and growth. A section of dorsal muscle was dissected for carbon (δ^13^C) and nitrogen (δ^15^N) stable isotope analyses. Muscle and invertebrate samples were dried (60°C) for 48 hours and later ground into a fine powder. A small amount (0.5–0.6 mg) of powder was encapsulated in tin cups. Water samples were collected from littoral (depths 0–3 m) and pelagic/profundal (0–33 m) habitats (Fig. 1) and directly stored in pre-combusted scintillation vials. Water samples for dissolved inorganic carbon were collected and directly injected into pre-phosphoric acid loaded and helium flushed Exetainer vials. All tissue and water samples were analyzed using an isotope mass spectrometer.

Whitefish were aged using both scales and otoliths [41]. According to these estimates of age, we selected a single individual of similar age (5+) from each morph to assess the lifetime thermal habitat use and respiration rates through otolith isotope analysis. Initially, we aimed to run several individuals per morph, but due to time and financial constraints this was not possible. However, of the majority of similar studies typically rely on small sample sizes and an individual-based approach without population-level comparisons [27], [32], [34]. Due to the lack of this type of data from other polymorphic poikilothermic populations, we hope that our preliminary results would promote more research on thermal adaptation.

One otolith of each morph was glued (Crystalbond©) to a microscope slide, sanded and polished flat on one side until the otolith nucleus was reached. Otoliths were photographed under a stereomicroscope, and the location and spacing of summer and winter circuli determined in order to characterize subsequent sampling paths via computer-controlled micromilling. This was conducted using a custom three-dimensional micromilling system in the Saskatchewan Isotope Laboratory facilities at the University of Saskatchewan [33]. This system permitted the sampling of 33–40 paths concordant with the growth banding in each otolith. The required sample size for isotope analysis (c. 7–25 µg) was obtained from almost all sampling paths (total 140 paths from 4 otoliths), with only four samples yielding insufficient material for analysis. Precision of carbon and oxygen analyses was 0.04 and 0.07‰, respectively.

### Temperature Calculations

Isotopic values for fish, otolith, invertebrate and water samples were calculated using:
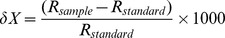
where *δX* is the stable isotope ratio of dorsal muscle or otolith carbonate and *R* is the isotope ratio of carbon, nitrogen or oxygen in the *sample* and the *standard*. As muscle isotope values show variations in C:N ratios, suggesting differences in lipid concentrations, we arithmetically corrected the values [42].

**Figure 2:**
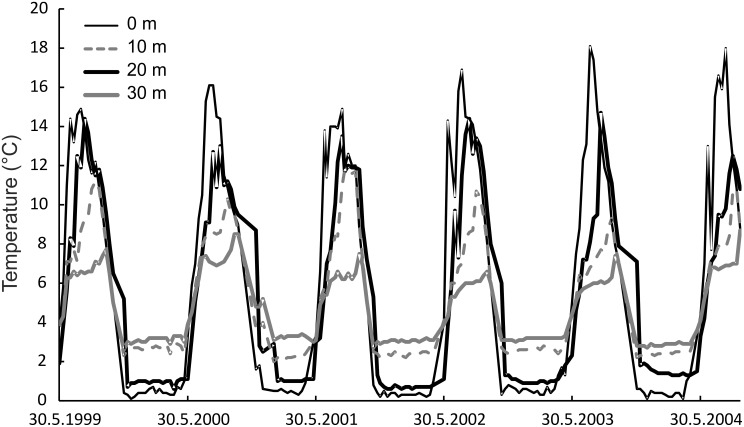
Annual temperature profiles (0–30 m) from Lake Inari, used as a proxy for Lake Paadar annual temperatures, during the life-time (1999–2004) of sampled whitefish morphs.



We used the average habitat-specific water and micro-milled otolith δ^18^O values to calculate whitefish lifetime thermal niche. Species-specific models are not available for whitefish: hence we estimated values using a general model [29] and models developed for closely related salmonids, brook charr [*Salvelinus fontinalis*; 43] and Arctic charr [*Salvelinus alpinus*; 44]. Here, the latter non-linear model by Godiksen et al. [44] for Arctic charr provided the most similar values compared to annual water column temperature datasets during the lifetime of sample fish from the same watercourse (Fig. 2). The other models gave on the average 0.5–5.0°C higher temperature values (Table S1) than the non-linear model for Arctic charr [44]. Otolith δ^18^O values reported relative to Vienna PeeDee Beleminite (VPDB) were transformed to the Vienna standard mean ocean water (VSMOW) scale:

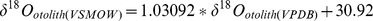



We used δ^18^O_water_ values from the water samples collected from each habitat type and whitefish otoliths to calculate the fractionation factors (*α*) for each sample:
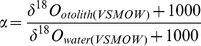
where *δ^18^O_otolith(VSMOW)_* is the isotope value of otolith samples and *δ^18^O_water(VSMOW)_* is the isotope value of water samples (both presented relative to the VSMOW scale). These values were then utilized in the polynomial model [44]:



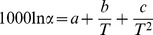
where *T* is temperature (10^3^/K), *a*, *b* and *c* are empirical constants. The empirical constants *a*, *b* and *c* were 1089.24, 89.9 and −617.19 [44].

### Respiration calculations

We used photographs to measure and calculate caudal aspect ratio, a morphometric index of fish swimming activity [45]:
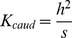
where *h* is the height of the opened caudal fin and *s* is caudal fin surface area. Here, higher values indicate streamlined and active fish such as pelagic cruisers, and low values represent less active, benthivores [45]. In general, *K_caud_* can be used successfully to predict otolith δ^13^C values [46]. Solomon *et al.* [31] further developed this relationship to directly estimate proportion of otolith carbon that is metabolically derived (*M*):



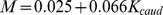



In addition, estimated of lifetime metabolism of the different whitefish morphs were calculated using the Wisconsin bioenergetics model with parameters for the generalized coregonid model [47]. To model parameters of consumption and to empirically estimate respiration, the model requires data that reflect lifetime temperature, growth in terms of wet mass, proportion of prey items consumed, as well as the energy density of whitefish morphs and invertebrate prey. For the input data, we used temperature estimates from the δ^18^O_otolith_ values, i.e. starting the modelling at the estimated time of hatching (1^th^ of June 1999, first modelling day = 150) and continued until the time of death (15^th^ of September 2004 = modelling day 2085). The age and length data from the total sample of each whitefish morph was used to calculate von Bertalanffy non-linear growth curves [48]:
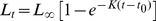
where *L_t_* is the expected total length at time *t*, *L_∞_* is the asymptotic total length (i.e. theoretical total length to which a fish would grow if time permitted), *K* is the rate at which the growth curve approaches the asymptote and *t_0_* is the hypothetical time at which *L_t_* was zero (i.e. hatching time).

The estimated lengths were transformed into wet mass with corresponding whitefish morph specific mass-length relationships [49]:
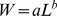
where *W* = wet mass (g), *L* = total length (cm), *a* = a constant, *b = *an exponent. Due to a lack of seasonal diet data, we assumed that all whitefish morphs consumed zooplankton prey during the first two months of life and thereafter ate prey associated with their respective habitat, i.e. DR whitefish consumed pelagic zooplankton, SSR whitefish fed on profundal benthic macroinvertebrates and LSR whitefish ate littoral benthic macroinvertebrates [21], [25]. As LDR whitefish diet consists of both pelagic zooplankton and insect pupae or adults, we used 50% pelagic and 50% littoral diet proportions. We calculated the energy density (*Y*) of whitefish morphs and invertebrates using the proportion of elemental carbon (%) derived from the isotope analysis of dorsal muscle and invertebrates [50]:



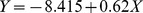
where *X* is the proportion of carbon (%). Energy densities of whitefish morphs and their invertebrate prey were kept as constant. The calculated energy contents for the SSR, LSR, LDR and DR whitefish were 5227, 5134, 5088 and 5150 J/g, respectively. Energy contents for littoral, profundal and pelagic invertebrates were 4694, 4341 and 2747 J/g.

The average gill raker count, gill raker length and isotope values of dorsal muscle (δ^13^C, δ^15^N) were statistically compared using analysis of variance and pairwise comparisons with Tukey’s test.

## Results

The total survey catch was 939 whitefish, of which 179 (40–50 individuals per morph) were subjected to more detailed analysis (Table 1). Whitefish morphs showed differences in gill raker number (ANOVA, F_3,175_ = 850, p<0.001) and the length of the longest raker (ANOVA, F_3,175_ = 208, p<0.001): the morphs differed from each other with regard to both measures (Tukey’s pairwise comparisons p<0.05), except SSR and LSR whitefish which overlapped in gill raker length. There were also significant differences in muscle tissue δ^13^C values (ANOVA, F_3,175_ = 167, p<0.001) and δ^15^N values (ANOVA, F_3,175_ = 200, p<0.001). Here, all morphs differed (Tukey’s pairwise comparisons p<0.05), except SSR and DR whitefish in δ^13^C values. The ecomorphological traits of the individuals selected for the otolith micromilling from each morph were in the range of values for the sample of the larger population (Table 1).

**Table 1:** Basic data from whitefish morphs (SSR = small sparsely rakered, LSR = large sparsely rakered, LDR = large densely rakered, DR = densely rakered whitefish).

Variable	SSR whitefish	LSR whitefish	LDR whitefish	DR whitefish
N	40	48	41	50
Total length (cm)	17.5±3.6 11.1–27.4 (16.7)	18.5±4.1 12.2–27.0(25.9)	29.3±1.9 21.6–32.1 (25.0)	14.0±1.5 11.3–17.8 (15.4)
Total mass (g)	48.1±39.610.8–218 (33.6)	52.9±36.4 13.0–160.0 (141.1)	202.9±32.8 85.3–252.6 (124.2)	20.2±6.2 10–37.7 (25.0)
Gill raker number	18.3±1.316–21 (19)	24.4±2.2 20–31(26)	33.7±1.9 30–38(33)	36.3±2.0 32–40 (38)
Gill raker length(mm)	2.4±0.51.5–4.0 (2.6)	2.7±0.6 1.6–4.3(3.4)	6.3±0.8 4.3–7.6(5.3)	4.1±0.6 2.8–5.4 (4.6)
δ^13^C (‰_VPDB_) dorsalmuscle	−28.4±1.1–31.9–26.8(−28.0)	−24.5±1.5–27.5–21.9, (−22.6)	−26.8±0.7–28.1–25.5 (−26.5)	−28.4±0.2–28.7–27.5 (−28.6)
δ^15^N (‰_AIR_) dorsalmuscle	9.9±0.49.0–10.8 (10.2)	7.1±0.6 6.0–8.1(6.6)	7.6±0.8 5.5–9.8(7.4)	8.6±0.5 7.8–9.9 (8.7)
Mean δ^18^O (‰_VPDB_)otolith	(−12.4)	(−12.6)	(−12.8)	(−12.8)
Mean δ^13^C_DIC_(‰_VPDB_) otolith	(−15.0)	(−11.6)	(−15.1)	(−16.1)
Invertebrate preyδ^13^C (‰_VPDB_)	−29.4±1.1	−23.4±1.9	−30.2±0.6,−23.4±1.9	−30.2±0.6
Invertebrate preyδ^15^N (‰_AIR_)	6.6±0.5	2.8±1.2	5.3±0.5, 2.8±1.2	5.3±0.5
*M*, estimated	0.260	0.286	0.322	0.349

The average (±SD) and range (min–max) for each morph are indicated with the individual used for the otolith micromilling in parenthesis. δ^18^O and δ^13^C_DIC_ values indicate the average value from all drilled transects. Isotopic values of invertebrate prey of LDR whitefish indicate both pelagic zooplankton and littoral macroinvertebrate values. *M* is the proportion of metabolically derived otolith carbon estimated from the caudal aspect ratio (*K_caud_*) [31].

**Figure 3:**
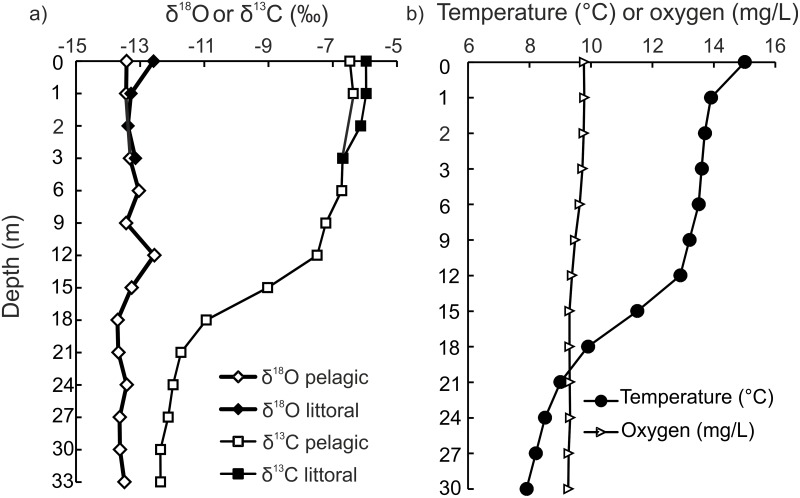
Oxygen (δ^18^O) and dissolved inorganic carbon (δ^13^C_DIC_) stable isotope values and profiles of temperature and oxygen (b) measured from the littoral (0–3 m) and pelagic (0–33 m) water column of Lake Paadar.

There was a clear variation in δ^18^O_H2O_ and δ^13^C_DIC_ values in the water column (Fig. 3). As expected, the highest δ^13^C_DIC_ values were observed near the surface (−6.4‰ _VPDB_) and the lowest at the deepest sampling depth of 33 m (−12.4‰_VPDB_). δ^18^O_H2O_ values were less variable, ranging between −13.7 and −12.6‰_VSMOW_ (Fig. 3). Mean δ^18^O_H2O_ values were calculated for each whitefish morph habitat i.e. littoral (mean for depths 0, 1, 2, 3 m, −13.1‰_VSMOW_), pelagic (mean for depths 0, 1, 3, 6, 9 m, −13.3‰_ VSMOW_) and profundal (mean for depths 21, 24, 27, 30, 33 m, −13.6‰_ VSMOW_). At the time of sampling, there was evidence for water column thermal stratification, with water temperatures ranging from 15°C at the surface to 8°C at 30 m (Fig. 3). Oxygen concentrations at this depth interval were *ca.* 9 mg l^−1^.

**Figure 4:**
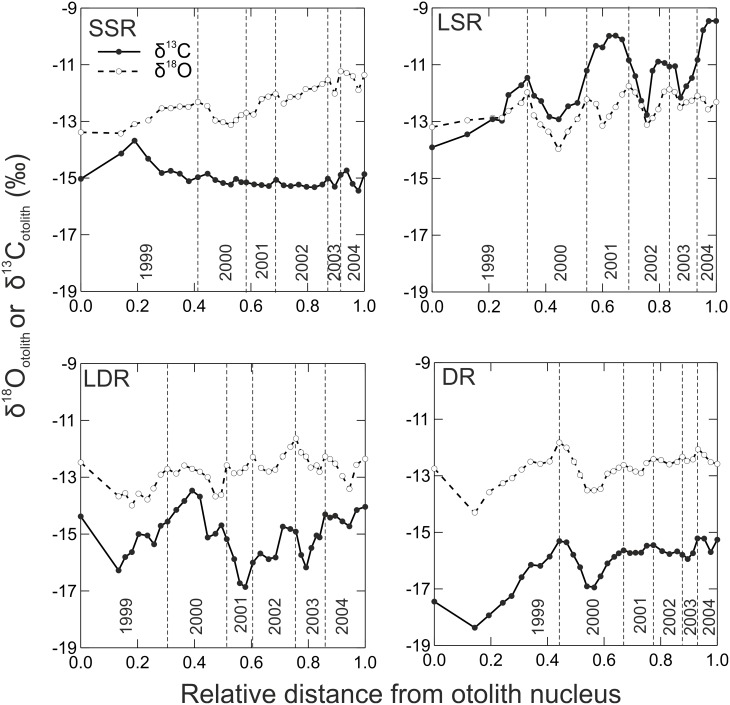
δ^18^O and δ^13^C values estimated from individual whitefish morph otoliths (SSR = small sparsely rakered, LSR = large sparsely rakered, LDR = large densely rakered and DR = densely rakered whitefish). The first x-axis value indicates the nucleus.

δ^18^O_otolith_ and δ^13^C_DIC_ values of otoliths indicated differences among the whitefish morphs (Fig. 4). The large bodied littoral LSR whitefish had consistently higher otolith δ^13^C values than the other morphs and also displayed the most pronounced seasonal variations. The individual of the pelagic LDR whitefish morph also exhibited strong seasonal variation in otolith δ^13^C values, as well as some indication of higher otolith δ^13^C values with age. The DR whitefish showed seasonal variation in otolith δ^13^C and δ^18^O values within the two first years of its life, but not in subsequent years. In contrast to the other morphs, the profundal SSR whitefish had the most stable otolith δ^13^C and δ^18^O values, exhibiting variation in only the first year of life (Fig. 4).

**Figure 5:**
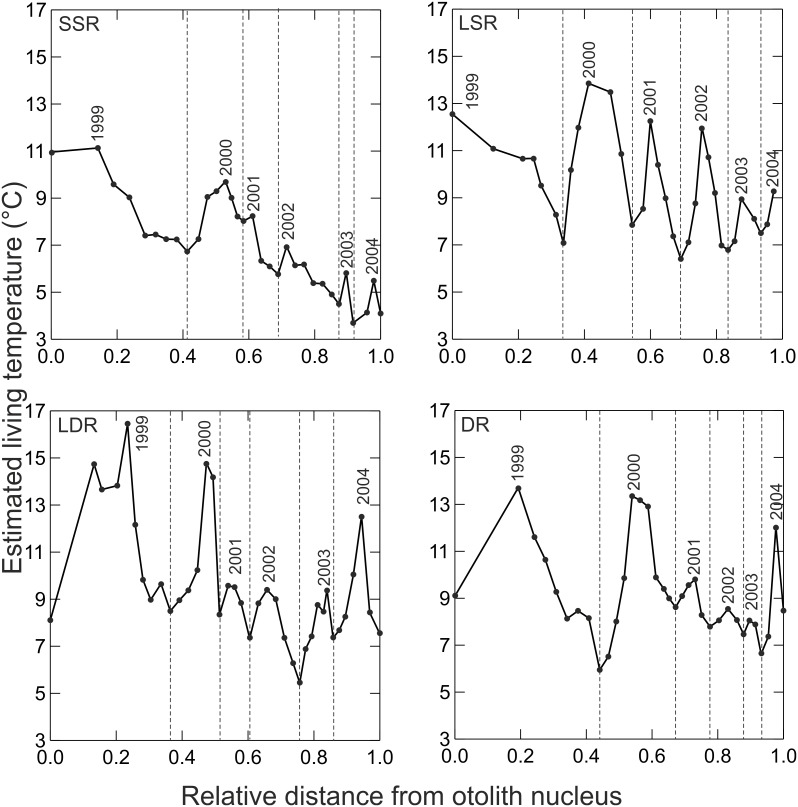
Lifetime temperature estimates for the sympatric whitefish morphs (SSR = small sparsely rakered, LSR = large sparsely rakered, LDR = large densely rakered and DR = densely rakered whitefish) according to stable isotopes of oxygen in otoliths. The first x-axis value indicates the nucleus i.e. estimated temperature at hatching.

δ^18^O_otolith_ values indicated seasonal water temperature variability for the 5+ individuals examined of each of the four of the whitefish morphs, which taken together, represents a time period that extended across six summer and five winter seasons (Fig. 5). Differences between summer and winter habitat temperatures were the most pronounced for the littoral LSR and the pelagic LDR whitefish, suggesting that both inhabit near-surface habitats. The pelagic DR whitefish display pronounced seasonal differences in the two first years of life, with apparently reduced seasonality in subsequent years. The profundal SSR whitefish individual displayed the most distinct thermal history, living at temperatures <11°C throughout their entire lifetime and with a pattern of decreasing temperatures after the first two years of life (Fig. 2 and 5). Although ontogenetic temperature niche shifts were most pronounced for the profundal SSR whitefish and the pelagic DR whitefish, there was a sign of decreased temperature variation for both the LSR and LDR whitefish too. δ^18^O values of the kernel (nucleus of otoliths), indicated that the two benthic morphs (profundal SSR whitefish and littoral LSR whitefish) hatched at higher temperatures (11–12°C) than the pelagic (DR and LDR whitefish) morphs (8–9°C).

**Figure 6:**
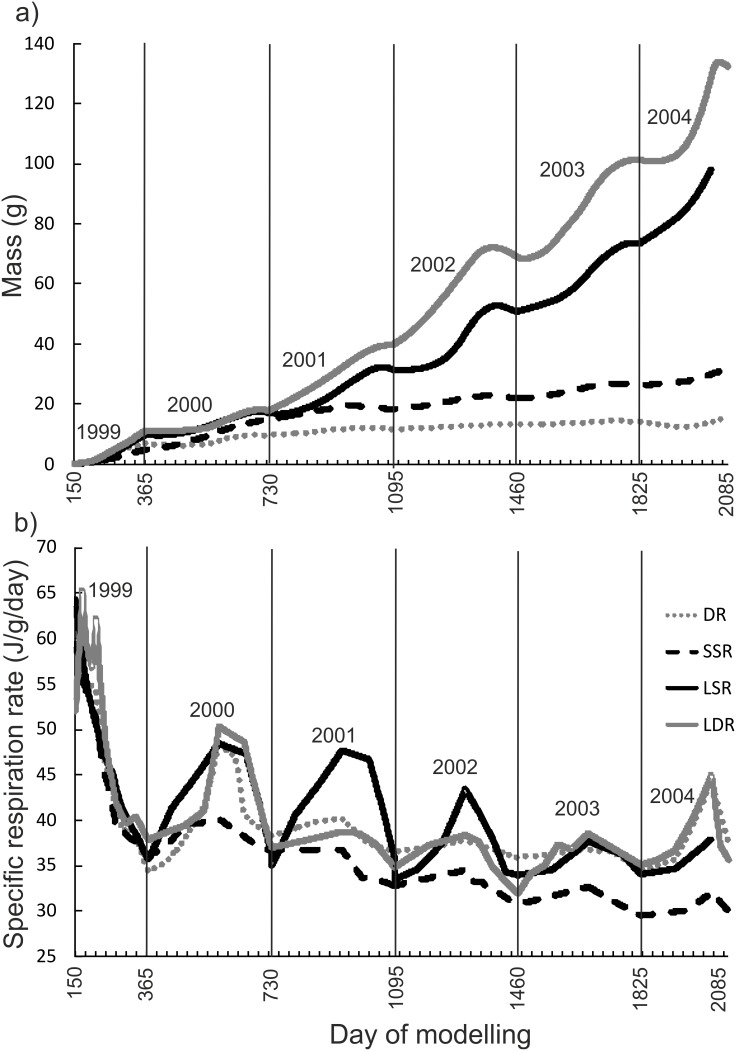
Bioenergetic modelling results of the weight gain (a) and the specific respiration rate (b) of whitefish morphs (SSR = small sparsely rakered, LSR = large sparsely rakered, LDR = large densely rakered and DR = densely rakered whitefish) from hatching (1.6.1999 i.e. day 150) to death (15.9.2004 i.e. day 2085). Years are separated with vertical lines.

The estimated proportion of metabolically derived otolith carbon (*M*) calculated from caudal fin morphology was the lowest for profundal SSR whitefish and the second lowest for littoral LSR whitefish (Table 1). The pelagic morphs had markedly higher *M*, with peak values observed for DR whitefish. Bioenergetic modelling revealed clear growth differences among the morphs. Somatic growth (assessed with wet mass in the model) was the fastest for the large bodied morphs (pelagic LDR whitefish and littoral LSR whitefish), both of which continue growing after six growing seasons (Fig. 6). Somatic growth was much lower for SSR whitefish, which displayed steadily increasing weight. The lowest growth was observed in the pelagic DR whitefish, which attained their estimated maximum size after three years of life. The corresponding estimates of specific respiration rates indicated that seasonal fluctuations were the highest for littoral LSR whitefish and the lowest for profundal SSR whitefish (Fig. 6). The specific respiration rates of pelagic LDR whitefish and DR whitefish were similar, and seasonal fluctuations were generally lower than those estimated from the littoral LSR whitefish.

## Discussion

We found support for our predictions that the different whitefish morphs occupied distinct thermal niches associated with differences in estimated respiration rates. The profundal SSR whitefish occupied the coldest, most stable thermal niche and had the lowest respiration estimates, whereas annual fluctuations in the thermal niche were the most pronounced in the littoral LSR whitefish. The thermal niche of the pelagic LDR and DR whitefish also showed clear seasonal fluctuations: respiration rates in individuals from these morphs were generally higher than the SSR whitefish and lower than the LSR whitefish. All morphs used a warmer thermal niche earlier in their ontogeny relative to later life, suggesting ontogenetic movement into colder water. Finally, we demonstrated that pelagic morphs hatched at lower temperatures than benthic morphs, according to the first otolith measurement in the nucleus, which likely relate to temporal and spatial differences in spawning.

In general, our evidence for thermal niche segregation among whitefish morphs provides a further dimension to previously documented ecomorphological and life-history divergence of these whitefish morphs [12], [21], [25]. Sympatric whitefish morphs have diverged and now exploit the three principal habitat types found in lakes (littoral, pelagic and profundal). LSR whitefish are ubiquitous to lakes in the study area, and when found in sympatry with other morphs is a littoral benthivore [21], [41]. Otolith δ^18^O values indicated that LSR whitefish tolerated pronounced yearly temperature variability, typical for shallow water habitats, suggesting a life-time occupation of littoral habitats. The results from the other benthivorous morph, the SSR whitefish [41] indicate consistent occupation of cold-water profundal niches. Both pelagic morphs displayed evidence for annual variability in their thermal niche, typical for thermally labile pelagic habitats. Results from the LDR whitefish showed more pronounced fluctuations than those shown by the DR whitefish, most likely related to near surface niche occupation in the LDR rather than the DR whitefish [25], [51]. In case of whitefish morphs, thermal adaptations are correlated to other ecomorphological divergence, which may be the case with other taxa as well.

There is some recent evidence that adaptive benefit of larger bill size of birds in warm environments is a metabolic benefit via heat loss [52]. Traditionally, bill size is very well known to correlate with foraging, song and mate selection for example in Darwin’s finches [6], [53]. Different *Anolis* lizard species in the Caribbean Islands utilize distinct parts of trees, reflecting different microclimates [7]. Thermal preferences may have at least partly originated via use of these microclimates and furthermore, may influence relative ecomorphological performance in their respective niche. It is apparent that ecomorphological and thermal adaptations can both work in the same direction and it is therefore difficult to judge which is more important with regard to the origin of divergence.

Resource competition is considered as an obvious driver of initial divergence of morphs in general [1], [54], [55] and could be followed by physiological divergence at a later stage. The different habitat and thermal niche selection may result from resource competition among whitefish morphs, in which pelagic zooplankton prey are more efficiently consumed by those pelagic morphs with more gill rakers compared to the sparsely rakered benthic morphs [23]. Previous evidence from allopatric populations has indicated that in the absence of DR whitefish, the pelagic habitat is frequently occupied by LSR whitefish [21], [56]. The strong ecomorphological divergence [21 and this study] among the morphs may also suggest physiological adaptations to different thermal niches. For example, in vendace morphs, the deeper water morph is clearly adapted to colder water, with a lower respiration rate relative to that of the shallow water morph [16]. An alternative scenario, especially in allopatric speciation and in the case of secondary contact [57], suggests that physiologically divergent species/morphs colonize and segregate the niches in the same lake or island according to their thermal preferences.

The SSR whitefish inhabiting cold profundal lake habitats exhibited the lowest estimated respiration rates according to calculations based on both morphology and bioenergetics. This is supported by differences in lifespan, as the profundal morph commonly lives for more than 20 years [58]. More variable metabolic rates were calculated for littoral LSR whitefish, which according to morphology (*M*) had the second lowest respiration rate. Both LSR and SSR whitefish forage on benthic macroinvertebrate prey [41], which requires the detection and capture of prey hiding in sediment. Benthic foraging behaviour is less energetically expensive than pelagic foraging, which relies on constant swimming to find zooplankton [45]. However, bioenergetic modelling indicated that the specific respiration rate of LSR whitefish, living in the habitat with the greatest temperature variability, was the highest. The difference between methods likely reflects the strong influence of living temperature on the results of bioenergetic modelling results. Similar differences between approaches were seen in both pelagic morphs: these had higher respiration rates than benthic morphs according to morphology, but were intermediate according to bioenergetics.

Thus, the most consistent finding is that profundal SSR whitefish have the lowest respiration rate, likely related to cold thermal habitats and the lowest risk of predation in profundal zones [59]. In contrast, pelagic morphs are subject to continuous risk of predation by piscivorous brown trout [40], [60]. The risk of predation by brown trout is reduced by diel vertical migration by DR whitefish [51], which may drive the highest estimated respiration rate for any of the four morphs. The pelagic DR morph also has the shortest average lifespan, rarely living more than six years [61]. Higher metabolic rates have been estimated for pelagic than benthic morphs through bioenergetic modelling in the closely related lake whitefish (*Coregonus clupeaformis*) [62]. Also, recent genetic results of lake whitefish indicate that metabolic traits are inherited and the pelagic morph consistently exhibits over-expression of genes related to metabolism and oxygen transport compared to benthic morphs, related to higher swimming activity required in pelagic habitats [63], [64]. There is an urgent need to evaluate the metabolic differences in other diverging taxa. Reptile and amphibian diversity is correlated with temperature, but very little is known about actual niche-specific metabolic adaptations [7], [65].

Individuals of each of the whitefish morphs examined here used warmer water layers during the summer in the first two years of life. This has been observed for many fish species in the first year of life, in both field and experimental studies [35], [66]. Dufour *et al.* [32] studied peri-alpine whitefish otoliths using the same techniques as used here and found that whitefish occupied warmer habitats during the first year of life. Whitefish larvae are known to concentrate in near shore areas after hatching and soon start to feed on small zooplankton [67]. Their diet may subsequently shift towards benthic macroinvertebrates or they may continue to consume zooplankton, depending on the individual lake and whitefish morph [68]. In nearby Lake Muddus, surface trawl catches in early autumn frequently contain 0+ DR whitefish in the pelagic habitat, whereas in sheltered shallow littoral bays 0+ LSR whitefish are caught (K. Kahilainen pers. obs.). To date, no 0+ SSR whitefish have been captured during these surveys, suggesting that a shift to morph-specific niches may occur during the first year of life. After the first two summer seasons, all morphs apparently choose cooler waters, shifting to their adult thermal niche. All the specimens studied here had reached or were very close to reaching sexual maturity, which occurs at different ages and sizes at the population level. Here, DR whitefish reach sexual maturity at the lowest age (2–3 years old), SSR whitefish at the highest age (6–7 years old) and the two other morphs at an intermediate 5–6 years old. Little is known about the exact spawning times and places for the different morphs, but field observations of spawning shoals in shallow water close to the main basin during field sampling in September 2004 indicated that DR whitefish were the first morphs to spawn in Lake Paadar. Observations in maturation and gonad state from the other morphs suggest that LDR and LSR whitefish spawned after the DR whitefish, with the SSR whitefish spawning last (K. Kahilainen pers. obs.). In coregonids, such inter-morph differences in spawning times are important factors driving reproductive isolation [17], [19], [63].

The general differences in spawning time shown by pelagic and benthic morphs may be indirectly related to the role of temperature governing the availability of invertebrate prey resources: pelagic zooplankton are only available in large amounts for a short period each summer, whereas benthic invertebrates are more consistently available year round [22], [41], [61]. The late spawning time shown by SSR whitefish may reflect temperatures in profundal habitats, which are the highest during the autumnal turnover of the water column. Physiological adaptation to a particular thermal regime may also be an important component of reproductive isolation in many species, but our current understanding is very limited [4]. Recently, niche segregation along thermal gradients associated with variation in both altitude and latitude has been shown to be efficient reproductive isolation barriers even in face of gene-flow in birds, amphibians and fish [69]–[71].

A recent modelling study also suggests that such physiological adaptations to different thermal habitat may be important in the formation of reproductive isolation and subsequent speciation [72]. Here, adaption to divergent thermal niches is likely to be of crucial importance to the relative capacity to assimilate the energy required to reach sexual maturity and develop gonads. If fish morphs are physiologically adapted to their individual thermal niche, this may cascade affecting spawning times and furthermore hatching times of eggs. The onset of spawning in whitefish is influenced by both temperature and light regimes [73]. An adaptation to lower temperatures may delay spawning times, as observed here for SSR whitefish, the most morphologically and genetically distinct morph [21], [37]. The isotope values from the otolith nucleus suggest potentially different hatching temperatures for benthic and pelagic morphs, perhaps related to either temporal or/and spatial differences in spawning. In experiments on lake whitefish morphs, hatching time was demonstrated to be genetically determined, with very small differences between pelagic and benthic morphs [63]. However, little is known about the early development of polymorphic fish eggs in nature, especially with regard to potential differences of degree-day counts among the morphs in relation to their physiological adaptations. These are potentially important thermal adaptations, as in amphibian thermal adaptations in early larval development that apparently separate different populations [71].

Our study indicates that otolith δ^18^O and δ^13^C analysis provide an excellent tool for understanding lifetime thermal niche and respiration values of whitefish morphs, when δ^18^O_H2O_ and δ^13^C_DIC_ values are obtained from lake water. Our preliminary study evaluates patterns based on snap shot sampling of water column during the summer stratification, whereas seasonal sampling should be done to track annual differences in δ^13^C_DIC_ values [32]. The results also verified the age determination estimates made previously from the same otoliths. Although, larger sample sizes are needed to reveal within-morph variation in thermal niche and respiration between multiple individuals, we hope that our preliminary results based on individual fish promote more studies on thermal adaptation from other polymorphic populations. Even though fine micromilling techniques were utilized to get 33–40 samples from each otolith, the resolution decreased due to declining fish growth rates after the first two years of life. At the very beginning of an individual fish’s life, it is unclear at which point during the egg development and what rate otolith growth starts. Reflecting this, do the first measurements taken from the otolith in nucleus indicate hatching temperatures or combine temperatures from the late egg development and hatching periods? Despite the lack of a clear answer, we have observed differences among the morphs, indicating distinct thermal habitats at the very beginning of their lifecycles.

All whitefish morphs can be considered as slow growing fish when compared to the fast growing marine fishes, such as cod (*Gadus morhua*) and halibut (*Hippoglosus stenolepis*) previously examined in similar studies [34], [36]. The estimated winter temperatures from otoliths are clearly too high as water temperatures in winter are usually <4°C even in the deepest parts of subarctic lakes [22]. This error is most likely due to the extremely small winter increments in the otoliths and subsequent integration of autumn and spring otolith growth into the same sample. Alternatively, these fish may have a shutdown temperature <4°C that precludes any otolith accretion during the cooler season. Otolith carbonate also records metabolism with winter rings clearly visible in whitefish, including the individuals analysed here. Based on difficulties in estimating timing and duration of a potential winter break in otolith growth and thus thermal estimation, we decided to present the full data in our results and discuss here the uncertainties involved. In addition, it should be noted that we used a fractionation equation developed for Arctic charr, which is adapted to colder temperatures than whitefish [44], [74]. An experimental approach using whitefish in general or even whitefish morphs is urgently needed to build species or whitefish morph specific equations between otolith δ^18^O values and temperature.

To conclude, we found a clear segregation of thermal niches between profundal and non-profundal whitefish morphs, but our capacity to differentiate between pelagic and littoral morphs was less successful. However, all individuals of the four whitefish morphs displayed evidence for ontogenetic thermal niche shifts, from warm juvenile summer habitats to cooler temperatures. Interestingly, estimated respiration rates were clearly correlated to foraging ecology and life-history, where the profundal SSR whitefish has lower rates than other morphs. These results also provide a useful starting point to conduct further studies of thermal niche life-histories with other polymorphic populations.

## Supporting Information

Table S1 **Summary table of temperature, oxygen, isotopes and bioenergetics values of study.**
(XLSX)
